# Supplementary material for: Sperm-Cultured Gate Ion-Sensitive Field-Effect Transistor for Non-Optical and Live Monitoring of Sperm Capacitation
Source: Sensors (Basel). 2019 Apr 14;19(8):1784. doi: 10.3390/s19081784 (PMC6515096; doi:10.3390/s19081784)
Supplement: Supplementary file 1 [file sensors-19-01784-s001.pdf]

sensors

Supplementary Materials

Sperm-Cultured Gate Ion-Sensitive Field-Effect Transistor for Nonoptical and Live Monitoring of Sperm Capacitation

Akiko Saito and Toshiya Sakata [1]

Department of Materials Engineering, School of Engineering, The University of Tokyo, 7-3-1 Hongo, Bunkyo-ku, Tokyo 113-8656, Japan
* Correspondence: sakata@biofet.t.u-tokyo.ac.jp

S1. Source-follower circuit for FET real-time measurement

In this study, the change in surface potential was monitored using the source-follower circuit shown in Figure S1 (Sakata et al., 2005). Here, V_G and V_D were set to constant values and V_S was controlled at a constant I_{DS}.

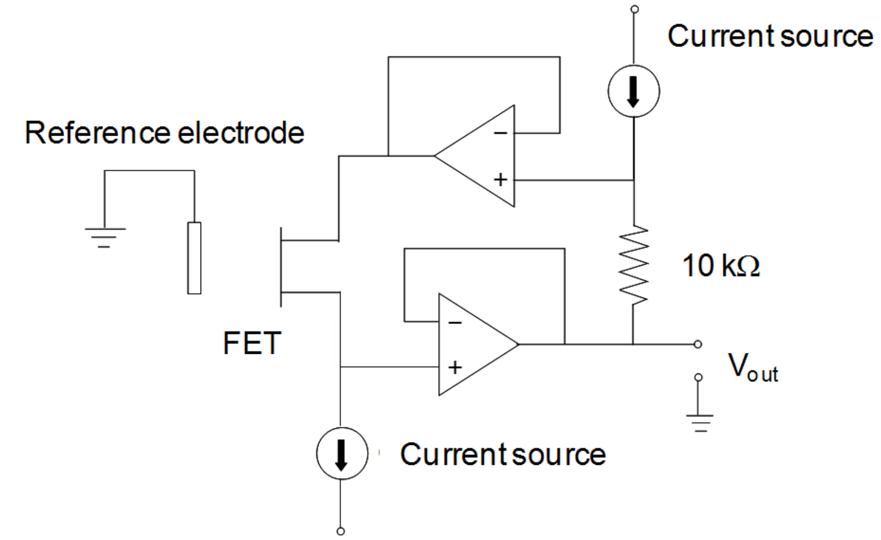

Figure 1. Electrical circuit. The change in surface potential (ΔV_{out}) at the gate was measured at a constant I_D (700 µA) and V_D (2.5 V) using the source follower circuit.

Reference

1. Sakata, T.; Kamahori, M.; Miyahara, Y. DNA analysis chip based on field effect transistors. *Jpn. J. Appl. Phys.* **2005**, *44*, 2854–2859.

S2. Estimation of sperm metabolism

To estimate the pH of the HTF medium buffer including HCO_3^-, the Henderson–Hasselbalch equation is employed for the bicarbonate buffer system as follows.

$$CO_2 \parallel_{liquid} + H_2O \rightleftarrows H_2CO_3 \rightleftarrows H^+ + HCO_3^-$$

$$\Rightarrow CO_2 \parallel_{liquid} + H_2O \rightleftarrows H^+ + HCO_3^-$$

(the ratio of $[H_2CO_3]$ to $[CO_2]_{liquid}$ or $[HCO_3^-]$ is assumed to be small)

$$pH = pKa' + log\frac{[HCO_3^-]}{[CO_2]_{liquid}}$$

Here, $[CO_2]_{liquid}$ is derived as 0.0307 (solubility coefficient) $\times P_{CO_2}$ (partial pressure of carbon dioxide) and $[HCO_3^-]$ is 25 mM. P_{CO_2} is calculated as 760 mmHg × 0.05, corresponding to 5% of carbon dioxide in the incubator system. pKa' indicates $pKa\ (dissociation\ constant) \times [H_2O]$, which is given as 6.095 (Tanemoto, 2018). Considering the change in pH from 7.4 to 7.2 shown in Figure 5b, the amount of carbon dioxide released from spermatozoa in the glass ring (1 mL), which corresponds to the increase in carbon dioxide, was calculated to be about 3% (1.2 mM), which was caused by sperm respiration, although the increase in $[CO_2]_{liquid}$ was calculated as 0.0307 × 760 mmHg × 0.03 (3%). Then, [ATP] in the glass ring was estimated to be about 7.5 mM on the basis of the following reaction.

$$C_{12}H_{12}O_6 + 6O_2 + 6H_2O \rightarrow 6CO_2 \|_{released} + 12H_2O + 38ATP$$

Reference

1. Tanemoto, M. Diagnostic use of base excess in acid–base disorders. *N. Engl. J. Med.* **2018**, *379*, 494–496.
